# Considerations in Adolescent Use of the Etonogestrel Subdermal Implant: A Cohort Study

**DOI:** 10.3389/frph.2021.780902

**Published:** 2021-12-23

**Authors:** Y. Frances Fei, Yolanda R. Smith, Melina L. Dendrinos, Monica W. Rosen, Elisabeth H. Quint

**Affiliations:** ^1^Section of Pediatric and Adolescent Gynecology, Nationwide Children's Hospital, Columbus, OH, United States; ^2^Department of Obstetrics and Gynecology, University of Michigan, Ann Arbor, MI, United States

**Keywords:** abnormal uterine bleeding, contraception, menstrual suppression, etonogestrel contraceptive implant, etonogestrel implant, dysmenorrhea, nexplanon, side effects

## Abstract

**Objectives:** To describe bleeding patterns and other side effects in adolescent implant users and characterize their impact on early discontinuation of the implant.

**Study Design:** This is a retrospective cohort study of female patients under 18 years who had an implant placed from 2013 to 2018. Data were collected on demographics, medical history, and side effects.

**Results:** Of 212 adolescents, the average age at insertion was 16 years and 84% desired placement for contraception. Common side effects included AUB (80%), mood changes (10%), and perceived weight gain (9%). Most (76%) used the implant for at least 12 months. Average time to removal was 22.1 months (SD 13.0 months) and this did not depend on presence of side effects. Twenty-seven percent of teens were able to achieve amenorrhea. Adolescents with frequent or prolonged bleeding were more likely to have implant removal prior to 12 months than those with other bleeding patterns (*p* = 0.003). Early removal was also more common in girls reporting weight or mood issues than those who did not (*p* < 0.001 and *p* = 0.045, respectively). BMI increased in 64% of adolescents. Average percentage change in BMI was 3.2% (0.87 kg/m^2^). There was no difference in baseline use of any mood-modulating medications in patients who did and did not complain of mood side effects following implant placement (*p* = 0.801).

**Conclusion:** Characterization of bleeding patterns following implant placement in adolescents have not previously been reported. Prolonged or heavy bleeding, mood issues, and perceived weight gain were associated with earlier removal of the implant. A relatively small number had early removal of the implant due to weight or mood complaints. Therefore, a history of obesity, depression, or other mood disorders should not be a deterrent to implant placement.

## Introduction

About 1–3% of adolescents in the United States use the etonogestrel subdermal contraceptive implant (hereafter “implant”) for contraception, similar to the general female population (1, 2). The American Academy of Pediatrics (AAP) recommends long-acting reversible contraception (LARC) as the first-line contraceptive choice for adolescents (1, 3). The implant and other hormonal contraceptives also offer non-contraceptive benefits, including menstrual suppression, premenstrual syndrome, and treatment of dysmenorrhea and/or endometriosis, but small studies have reported low interest in these benefits in adolescents (1–2%) (4, 5).

In the CHOICE project, which promoted LARC use in women of all reproductive ages, continuation rates of contraceptives were much higher in LARC users compared to non-LARC users (67.2 vs. 31.0%), which is important in pregnancy prevention. However, implant users had only a 56.2% rate of continuation at 3 years [compared to almost 70% for intrauterine devices (IUD)]. This lower rate of continuation is likely due, in part, to a less favorable bleeding profile compared to IUD (6).

Abnormal uterine bleeding (AUB) is reported in up to 78% of all implant users (7). Data in adolescents is lacking but, in a few small studies of adolescents using implants, 43–61% reported bothersome bleeding. Despite this, studies on adolescents have reported 75–80% continuation rates at 1 year (1, 4). AUB can improve over time, with about 50% of unfavorable bleeding patterns improving over the first 6–12 months in adults (7–9). Other common side effects reported in the general population include weight gain (12%), mood changes (1–5%), and acne (10–14%), though their prevalence in adolescents have not been well-studied (2, 10).

Very few studies focus on outcomes associated with implant use in adolescents and reasons for discontinuation. At the time of writing, there were no other studies describing prevalence of specific bleeding patterns following implant placement. This study aimed to describe outcomes of implant use in adolescents, including bleeding patterns and other side effects. The secondary goal of this study was to describe the impact of specific bleeding patterns and side effects on time to discontinuation of the implant.

## Methods

This is a retrospective cohort study of female patients younger than 18 years who had implant placement at any Michigan Medicine outpatient clinic from January 2013 to December 2018. Patients were identified using Current Procedural Terminology (CPT) codes 11981–11983 for “insertion or removal with reinsertion of biodegradable drug delivery implant.” Patients who did not have at least 12 months of follow-up visits and no documentation of early removal of the implant were excluded. Early implant removal was defined as removal prior to 12 months. This study was approved by the Institutional Review Board at the University of Michigan.

Electronic medical records were reviewed and data were collected on demographics, medical history, reason for implant, timing of placement, provider specialty, treatment of bothersome bleeding, side effects, reasons for removal of implant, and time to removal. Bleeding patterns following implant placement were categorized according to World Health Organization definitions. Amenorrhea was defined as no bleeding or spotting, infrequent as <3 bleeding or spotting episodes, monthly as 3–5 episodes, frequent as more than 5 episodes, and prolonged as any one episode lasting longer than 14 days, all in relation to the previous 3-month period (11). Any bleeding pattern other than amenorrhea was considered AUB.

Medications with potential effects on AUB or mood were categorized by type. Selective serotonin reuptake inhibitors (SSRI) and valproic acid can affect clotting efficacy, so were grouped separately (12–14). Enzyme-inducing anti-epileptic drugs (EI-AED) can affect metabolism of hormonal medications and were also considered separately (15). We used the term “mood-modulating medications” to include SSRI, tricyclic anti-depressants, anti-psychotics, and anticonvulsants when specifically used for mood disorders.

Weight calculations were based on body mass index (BMI) at time of insertion compared to time of removal or 12 months after placement, whichever came first. Patients who were immediately postpartum were not included in the weight calculations.

Descriptive statistics and frequency tables were used to summarize the data. Chi-square and Fisher's exact tests were performed to compare categorical variables. Analysis of variance tests were used to compare continuous variables. Levene's test was used to assess for equality of variances. SPSS Statistics version 26 (IBM) was used to perform statistical analyses.

## Results

### Demographics

A total of 212 patients were included in this study. The average age of menarche was 11.9 years (standard deviation [SD] 1.3 years) and average age at insertion was 16 years (SD 1.2 years, range 12–17 years). The majority of patients were non-Hispanic white (Table 1). Almost 30% of adolescents (62/212) were on a mood-modulating medication at the time of insertion. Most patients (92.5%) were nulliparous, 66.0% had been sexually active prior to implant placement, and 84.0% desired the implant for contraceptive reasons (Table 2). Prior to insertion, 30.3% (43/142) of sexually-active adolescents reported barrier-only or no contraception. Almost half (50.5%) reported a history of dysmenorrhea and 5.2% reported cyclic mood symptoms, though these were not necessarily the reason for implant placement.

**Table 1:** Patient characteristics at time of implant placement (*n* = 212).

	***N***	**%**
**Race**		
Caucasian or White	146	68.9%
African American or Black	56	26.4%
Asian	2	0.9%
Native American or Alaskan Native	1	0.5%
Other	5	2.4%
Unknown	2	0.9%
**Ethnicity**		
Hispanic or Latino	8	3.8%
Non-Hispanic or Latino	201	94.8%
Unknown	3	1.4%
**Insurance type**		
Private	142	67.0%
Government	66	31.1%
None	4	1.9%
Medications at time of insertion^*^		
SSRI	47	22.2%
Tricyclic anti-depressants	6	2.8%
Anti-psychotics	13	6.1%
Valproic acid	3	1.4%
EI-AED	4	1.9%
Other anticonvulsants	5	2.4%

*CHC, combined hormonal contraceptives; DMPA, depot medroxyprogesterone acetate; LNG-IUD, intra-uterine device; SSRI, selective serotonin reuptake inhibitors; EI-AED, enzyme inducing anti-epileptic drugs*.

* *Participants could have more than one*.

**Table 2:** Factors at time of implant placement (*n* = 212).

	***N***	**%**
**Contraception immediately prior to implant**		
Barrier-only or no contraception	43	20.3%
Never sexually active	70	33.0%
CHC	45	21.2%
Oral progestin	9	4.2%
DMPA	27	12.7%
LNG-IUD	3	1.4%
Implant	1	0.5%
Recently postpartum^†^	9	4.2%
Unknown	5	2.4%
Reason for implant^*^		
Contraception	178	84.0%
Menstrual suppression	60	28.3%
Dysmenorrhea	37	17.5%
Cyclic mood symptoms	2	0.9%
Other	6	1.9%
**Days from LMP or date of delivery to day of insertion**		
≤7 days	53	25.0%
8–14 days	29	13.7%
15–21 days	27	12.7%
22–35 days	35	16.5%
>35 days	23	10.8%
Unknown	45	21.2%
**Implant placement specialty**		
Family medicine	43	20.3%
Obstetrics and gynecology	153	72.2%
Pediatric primary care	16	7.5%

*LMP, last menstrual period; LARC, long-acting reversible contraceptives, including etonogestrel subdermal implants and intrauterine devices*.

† *Recently postpartum was defined as within 4 weeks of delivery or 6 months of exclusive breastfeeding*.

* *Participants could have more than one*.

### Outcomes

The most common side effects included AUB (79.7%), mood changes (9.9%), and perceived weight gain (9.4%). These were also the most common reasons for early removal (Table 3). Despite AUB and other side effects, 162 (76.4%) used the implant for at least 12 months. Average time of removal did not depend on reason for placement, pregnancy history, sexual activity, type of insurance, baseline BMI, race/ethnicity, or age.

**Table 3:** Outcomes following implant placement.

	**Total *N* (%)**	**Implant removed <1 year**	**Implant in place ≥1 year**	***p*-value**
Total removed	147	50	97	
**Removal reason**				
Device expiration	48 (32.7%)			
Abnormal uterine bleeding	69 (46.9%)	34	35	<0.001
Weight changes	15 (10.2%)	12	3	<0.001
Mood issues	11 (7.5%)	7	4	0.045
Acne	5 (3.4%)	3	2	0.337
Foreign body intolerance	4 (2.7%)	1	3	1.000
Headaches	3 (2.0%)	2	1	0.267
Desires pregnancy	2 (1.4%)	0	2	0.548
Current pregnancy	1 (0.7%)	1	0	0.340
Unknown/other	9 (6.2%)			
**Next contraception**				
Continued pregnancy	1 (0.7%)			
Desired pregnancy	2 (1.4%)			
Barrier or abstinence	25 (17.0%)			
CHC	35 (23.8%)			
Progestin-only pills	4 (2.8%)			
DMPA	15 (10.2%)			
LNG-IUD	20 (13.6%)			
Copper IUD	1 (0.7%)			
Replacement of implant	44 (29.9%)			

*CHC, combined hormonal contraceptives; DMPA, depot medroxyprogesterone acetate; LNG-IUD, levonorgestrel intrauterine device; IUD, intrauterine device*.

Almost all patients (44/48) who had the implant removed for device expiration desired replacement of the implant. Two patients were found to be pregnant within 8 weeks following implant placement. The implants were placed on cycle day 19 and 22, respectively. Both patients were using barrier contraception only and had a negative UPT at insertion. One patient underwent a therapeutic abortion and left the implant in place for 3 years; the other patient had the implant removed and had a full-term live birth.

### Bleeding Patterns

Of the 212 patients included in the study, 169 (79.7%) reported AUB following placement. Twenty-seven percent of all patients with AUB eventually achieved amenorrhea (Table 4). On average, AUB was managed expectantly for 11.1 months (SD 10.8 months) prior to the patient desiring removal or trial of medication to treat AUB. Of 36 patients with confirmed use of medication treatment of AUB, 27.7% became amenorrheic with treatment. No method was markedly superior in treating AUB. Presence of AUB did not depend on timing of placement relative to last menstrual period, previous use of hormonal contraception, or use of mood-modulating medications. There was no difference in average baseline BMI or change in BMI in adolescents who did and did not experience AUB. However, at 12 months adolescents who reported AUB were more likely to have lower average BMI than those who did not have AUB (25.99 vs. 30.0 kg/m^2^, *p* = 0.013).

**Table 4:** Bleeding patterns following implant placement.

	**Total *N* (%)**	**Implant removed <1 year**	**Implant in place ≥1 year**	***p*-value**
**Final bleeding pattern**				
Amenorrhea	40 (27.0%)	7	33	0.003
Infrequent	18 (12.2%)	3	15	
Monthly	33 (22.3%)	2	31	
Frequent	32 (21.6%)	13	19	
Prolonged	25 (16.9%)	10	15	
Unknown	21 (12.4%)			
Total	212	50	162	

Average time to removal was 22.1 months (SD 13.0 months) and this did not depend on presence of AUB (*p* = 0.83). However, adolescents who had frequent or prolonged bleeding were more likely to have early removal of the implant than those with other bleeding patterns (*p* = 0.003; Table 4). In addition, those who reported AUB as a reason for removal were also more likely to have early removal than those who did not (*p* < 0.001; Table 3).

### Weight Changes

Average BMI at implant placement was 25.6 kg/m^2^ with an average percentage increase in BMI of 3.2% (0.87 kg/m^2^) over the first 12 months. Those who reported weight gain as a reason for removal were more likely to have the implant removed prior to 12 months than those who did not report this concern (*p* < 0.001; Table 3). There was no difference in BMI at time of insertion (28.17 vs. 25.39 kg/m^2^, *p* = 0.231) in those who did and did not report weight gain, respectively. Though not statistically significant, there was a trend toward higher BMI after 12 months in those who reported weight gain compared to those who did not (31.11 vs. 26.17 kg/m^2^, *p* = 0.055). In addition, those who reported weight gain experienced a 10.7% increase in average BMI and those who did not cite this concern experienced a 2.4% increase in BMI (*p* = 0.001).

Overall, 63.5% of girls experienced an increase in BMI during the study period, with an average increase of 8.3%. The remaining 36.5% had a decrease in BMI, on average 5.7%. There was no difference in average time to removal of the implant in those with an increase in BMI compared to those with a decrease in BMI (21.5 vs. 22.6 months, *p* = 0.668).

### Mood Disorders

Those who reported mood changes as reason for removal were more likely to have the implant removed prior to 12 months than those who did not (*p* = 0.045; Table 3). There was no difference in baseline use of any mood-modulating medications in patients who did and did not complain of mood side effects following implant placement (*p* = 0.801). About 30% of patients (6/21) complaining of mood side effects had a change in mood-modulating medications prior to implant removal; 5/6 were already taking an SSRI and added or changed medications, and 1/6 started a medication.

## Discussion

This large study on adolescent implant use provides new data on the typical adolescent experience. More than three-fourths of our cohort continued use for at least 1 year, with about 30% desiring replacement of the implant. AUB was the most common reason for removal, consistent with the literature, and we found that pattern of bleeding was an important determinant. Adolescents with prolonged or heavy bleeding were more likely to request removal prior to 1 year of use. Almost 30% achieved amenorrhea either with expectant management or medical treatment of AUB. Perceived weight gain was also frequently cited as reason for implant removal, though there was no difference in average BMI at time of insertion and after 12 months. About 10% of patients also reported mood changes, and there was no difference in baseline use of mood-modulating medications in those who did and did not report this side effect.

Unpredictable bleeding is very common following implant placement, seen in 75–80% of patients in this and other studies (8). Bleeding usually started soon after placement, yet adolescents waited an average of 11.1 months before pursuing implant removal or treatment of AUB. In our study, adolescents with AUB were more likely to have a lower average BMI at 12 months than those without AUB (25.99 vs. 30.0 kg/m^2^, *p* = 0.013). Studies have suggested that AUB is more common with higher serum etonogestrel concentrations and that lower BMI was associated with higher serum levels, which may explain the relationship seen in our study (16, 17). This could be related to increased stability of the endometrium due to increased endogenous estrogen levels in girls with higher BMI, similar to using exogenous estrogen to improve bleeding patterns related to endometrial atrophy (18).

In this study as well as others, AUB was the most frequently cited reason for discontinuation. One study reported up to 61% of adolescents requested removal prior to 12 months due to bleeding (4). In the current study cohort, 47% of patients reported AUB as reason for removal, but in those requesting removal prior to 12 months, 68% cited AUB as the reason. Early removal was more common in adolescents with frequent or prolonged bleeding episodes. Although data is limited due to lack of consistent documentation, we found that almost 30% of patients achieved amenorrhea with medical management of AUB. Therefore, treatment of AUB can help increase continuation of the implant, and if bothersome breakthrough bleeding remains present 3–4 months after placement, patients should be instructed to follow up for discussion of options.

Weight gain was the second most common reason for early removal. Similar to studies in the general population, 9% of patients in this study reported weight gain as a side effect (2, 3). One study of adolescent implant users reported no difference in weight between implant users and controls (which included users of other hormonal contraceptives) (19). Other studies have reported a slight increase in BMI in a general population of implant users, although there was no significant difference when compared to controls (20, 21). This study found an average of 3.2% increase in BMI (0.87 kg/m^2^) following implant placement, but no difference in average BMI between those who did and did not report weight gain. However, weight gain can be variable and unpredictable. For example, those who reported weight gain experienced an 11% average increase in BMI compared to those who did not report weight gain who experienced a 2% increase (*p* = 0.001). For comparison, the 50th percentile BMI increases about 0.7 kg/m^2^ per year in adolescents from age 12 to 17 years (the range of our study population) (22). Therefore, some of the perceived weight changes and changes in BMI are likely due to normal growth and development (20). Overall, about 64% of girls experienced an increase in BMI during the study period, on average 8.3%, though only 10% cited this as a reason for removal. Therefore, baseline BMI and concern for weight gain should not be a contraindication to implant use.

Adolescents reporting mood changes in our study were more likely to request early removal (*p* = 0.045). Mood effects from hormones, especially progesterone-only medications, have remained an understudied field with conflicting results (23–25). Some studies have found an increased risk for antidepressant use or diagnosis of depression in adolescents using hormonal contraception, especially progestin-only pills (23, 24). However, a recent systematic review found minimal association between use of progestin-only contraception and depression (25). No studies have reported specifically on mood changes in adolescent implant users, but studies of the general population have reported a 1–5% rate of mood changes related to implant use (26). In this study, about 10% of adolescents reported worsening mood issues and 7.5% cited this as a reason for removal. More than 20% of girls in this study were taking anti-depressants at the time of implant placement, a higher proportion than in the general adult population (13%) (27). However, there was no difference in baseline medication use between those who did and did not have complaints of mood changes. Therefore, a history of depression or other mood disorders should not be a deterrent to implant placement.

This is one of the largest studies on usage and outcomes of implant placement in an adolescent population with at least 12 months of follow up data. Characterization of bleeding patterns following implant placement in adolescents have not previously been reported. Limitations include the retrospective nature of this study, with reliance on the electronic medical record. Detail and accuracy of documentation of bleeding patterns at follow up visits likely differed amongst providers and could not be standardized as the data were collected retrospectively. Specific data from patients were not collected if documentation was lacking. Objective data on specific side effects, such as mood changes, were limited and unable to be compared with the baseline population of adolescents. Data on weight changes in adolescents were also difficult to generalize as no comparison group was available.

## Conclusions

Overall, we found that most adolescents chose to continue implant use beyond 1 year and many opt for replacement at time of device expiration. Almost 30% of adolescents achieved amenorrhea with either expectant management or medical treatment of AUB. Prolonged or heavy bleeding, mood issues, and weight gain were associated with earlier removal. Despite the large proportion of girls in this study with mood disorders, a relatively small number had early removal of the implant due to increased mood complaints. Therefore, a history of depression or other mood disorders should not be a deterrent to implant placement. Similarly, baseline BMI and concern for weight gain should not be a contraindication to implant use. Counseling on and treatment of side effects may increase length of implant use, leading to improved contraception.

## Data Availability Statement

The raw data supporting the conclusions of this article can be made available by the authors, without undue reservation.

## Ethics Statement

The studies involving human participants were reviewed and approved by University of Michigan Institutional Review Board. Written informed consent from the participants' legal guardian/next of kin was not required to participate in this study in accordance with the national legislation and the institutional requirements.

## Author Contributions

YF, YS, and EQ conceptualized the study. YF collected the data, performed statistical analyses, and wrote the first draft of the manuscript. All authors assisted in interpretation of the data, revised the manuscript, and approved the final version.

## Conflict of Interest

The authors declare that the research was conducted in the absence of any commercial or financial relationships that could be construed as a potential conflict of interest.

## Publisher's Note

All claims expressed in this article are solely those of the authors and do not necessarily represent those of their affiliated organizations, or those of the publisher, the editors and the reviewers. Any product that may be evaluated in this article, or claim that may be made by its manufacturer, is not guaranteed or endorsed by the publisher.
